# Cohort Differences in Neurobehavioral Symptoms in Chronic Mild to Severe Traumatic Brain Injury

**DOI:** 10.3389/fneur.2019.01342

**Published:** 2020-01-09

**Authors:** Shannon B. Juengst, Andrew Nabasny, Lauren Terhorst

**Affiliations:** ^1^Department of Physical Medicine & Rehabilitation, University of Texas Southwestern Medical Center, Dallas, TX, United States; ^2^Department of Occupational Therapy, University of Pittsburgh, Pittsburgh, PA, United States

**Keywords:** traumatic brain injury, behavior, emotion, education, health disparities

## Abstract

Our understanding of neurobehavioral symptoms after traumatic brain injury (TBI) largely relies on data gathered in studies conducted at academic medical centers or large clinical centers with research infrastructure. Though this often provides a well-characterized clinical sample, it may also introduce bias based on geographic locations served by these institutions and personal factors associated with patient access to these institutions. We collected neurobehavioral symptoms via the self-reported Behavioral Assessment Screening Tool (BAST) in a National TBI Cohort (*n* = 263) and a Medical Center TBI Cohort (*n* = 218) of English-speaking community-dwelling adults with chronic TBI. The primary focus of the present study was to compare demographics and neurobehavioral symptom reporting across the two cohorts and to discuss the implications of any such differences on interpretation of symptom scores. Across all BAST subscales (Negative Affect, Fatigue, Executive Function, Impulsivity, and Substance Abuse), participants in the National TBI Cohort reported significantly more frequent symptoms than those in the Medical Center TBI Cohort (*p*'s < 0.001). Participants in the National TBI Cohort were more likely to be non-White and Hispanic compared to the Medical Center TBI Cohort, and those with mild TBI in the National TBI Cohort were more likely to have less than a high school education than those with mild TBI in the Medical Center TBI Cohort. Individuals with TBI recruited through academic and clinical institutions may not be representative of individuals with TBI living across the United States.

## Introduction

Neurobehavioral symptoms, including aggression, disinhibition, lack of motivation, and difficulty planning/executing actions (1, 2), are common after traumatic brain injury (TBI) and adversely affect participation and quality of life even many years after injury (3–9). Race and/or ethnicity (10), education (11), and gender (12, 13) may also contribute to differences in neurobehavioral symptoms after TBI. Racial and ethnic minority groups report more psychiatric symptoms and cognitive deficits after TBI than non-Hispanic white individuals (10, 14–16). Racial and ethnic minorities also experience healthcare disparities after brain injury (14, 16–22) that can magnify the long-term consequences of injury (14, 23–25). A review by Arango-Lasprilla and Kreutzer (14) on the effects of racial and ethnic disparities on functional, psychosocial, and neurobehavioral outcomes after TBI concluded that, compared to non-Hispanic white individuals, individuals from racial and ethnic minority groups received lower-quality treatment and had worse functional outcomes. The authors suggest multiple potential mediating factors between race/ethnicity and poor outcomes, including socioeconomic status, quality (not just quantity) of education, access to care, quality of care, and transportation barriers (14). These factors are also all associated with where an individual lives. In the general population, geographic location (e.g., proximity to high quality medical care) is associated with symptoms, long-term outcomes, and health service utilization (26, 27). For individuals with TBI, those living in more rural areas, as compared to those living in more urban areas, have more pre- and post-injury comorbities and report more unmet service needs (28). Unmet needs may be the result of fewer rehabilitation professionals, services, and facilities available in rural areas (29).

Our understanding of neurobehavioral symptoms after TBI largely relies on data gathered in studies conducted at academic medical centers or large clinical centers with research infrastructure. Though this often provides a well-characterized clinical sample, it may also introduce bias based on geographic locations served by these institutions. Geographic location affects patient access to these institutions and is likely a proxy indicator of other factors related to healthcare disparities noted above. To determine whether neurobehavioral symptoms after TBI are associated with geographic location and different recruitment strategies, this study compared neurobehavioral symptoms from a nationally representative sample of adults with chronic TBI assessed anonymously to a sample of adults with chronic TBI recruited from multiple academic medical and clinical centers.

## Materials and Methods

### Setting

We collected neurobehavioral symptoms via a validated self-reported survey in two study cohorts of community-dwelling adults with chronic TBI. The first (National TBI Cohort) was a nationwide self-reported survey of community-dwelling adults with self-reported TBI collected electronically via Qualtrics™. The second (Medical Center TBI Cohort) was a combined data set of three separate studies including community-dwelling adults with a chronic history of documented TBI recruited through academic medical centers in major metropolitan areas and their surrounding communities. Study 1 was the first to pilot the BAST in community-dwelling adults with TBI (30). Study 2 was a randomized clinical trial of a healthy lifestyle weight loss intervention vs. education intervention for community-dwelling adults with TBI that collected the BAST during the baseline assessment (31). Study 3 piloted the BAST as part of the Concussion Network of North Texas (ConTex) research registry on concussion recovery.

### Participants

#### National TBI Cohort

Participants were adults (≥18 years old), fluent in English, with a self-reported history of TBI and no self-reported history of schizophrenia or dementia, who electronically consented to participate in this anonymous survey study.

#### Medical Center TBI Cohort

All participants were community-dwelling adults (≥18 years old), fluent in English, with a documented history of TBI. Specific inclusion/exclusion criteria for each of the three studies are as follows:

Study 1 participants were ≥18 years old and at least 3 months post moderate–severe TBI. Inclusion criteria were as follows: (1) documented complicated mild to severe TBI, (2) ≥3 months post-injury, (3) >18 years old, and (4) written English fluency. Exclusion criterion was inability to provide informed self-consent. We collected age, gender, race, education, time since injury, and the BAST via paper questionnaires mailed to study participants.
Study 2 participants were 18–64 years old and at least 6 months post moderate–severe TBI. Inclusion criteria were as follows: (1) 18–65 years old, (2)≥6 months post-injury, (3) moderate to severe TBI, (4) BMI ≥ 25 kg/m^2^, and (5) access to or willingness to use a smartphone. Exclusion criteria were as follows: (1) contraindicated health conditions, (2) non-English fluency, (3) non-community dwelling, and (4) taking diabetes medication.
Study 3 participants were those >20 years old who attended an initial clinic visit at an academic medical center clinic for a mild TBI (e.g., concussion). Inclusion criteria were as follows: (1) diagnosis of concussion presenting at a participating clinic, (2) visual acuity/hearing adequate to complete interviews and questionnaires, (3) English fluency, and (4) ability to provide informed consent. Exclusion criteria were as follows: (1) loss of consciousness >30 min, (2) known skull fracture or intracranial bleed, (3) spinal cord injury with SIA score of C or worse, and (4) most recent concussion occurring >6 months ago.

### Procedures

#### National TBI Cohort

**Figure 1:**
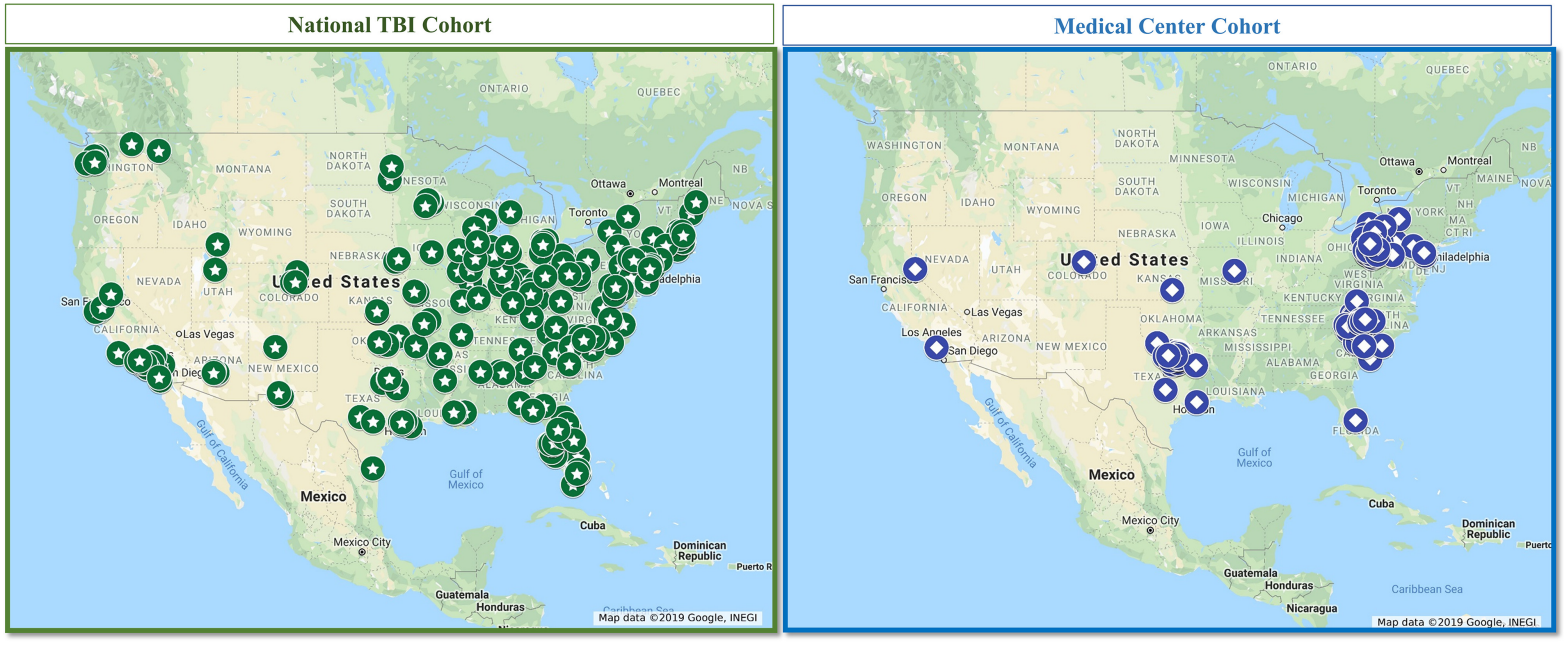
Map of participants from the National TBI Cohort and the Medical Center Cohort.

We collected all data using Qualtrics™ (Qualtrics, Provo, UT) online, HIPAA-compliant survey platform. We previously provided a detailed description of the data collection for this cohort and the methods to ensure integrity of the data collected (32). Briefly, we leveraged Qualtrics not only for the online platform but also for its capacity to serve as a survey panel aggregator to collect survey responses from a national sample. We confirmed TBI presence and severity with an electronic version of the OSU-TBI (33); prior studies indicate that collecting OSU-TBI data via electronic survey is valid (34). We generated a map of all responses to show national representation, depicted in Figure 1.

#### Medical Center TBI Cohort

*Study 1:* Adult participants with TBI were recruited through two academic medical centers' rehabilitation research registries and previous and ongoing research studies. We collected the demographic data and BAST, in addition to a number of other measures, via paper questionnaires. Documentation of TBI was available through medical record review and/or participation in previous research studies.

*Study 2:* Adult participants with TBI were recruited through an academic medical center, a major healthcare system, and the community in a large, metropolitan area. We collected demographic data (age, gender, race, ethnicity, and education) at baseline via interview and the BAST at baseline via electronic survey in RedCap™.

*Study 3:* ConTex recruits individuals age 5 and over from local concussion clinics in the North Texas area; for the purposes of this study, we included only adults >20 years old, as an adolescent version of the BAST is collected in ConTex for individuals 12–20 years old. We collected the BAST as part of the 3 month post-initial clinic visit follow-up assessment completed electronically via REDCap™.

*Full Medical Center TBI Cohort:* We generated a map of all participants in the Medical Center TBI Cohort, depicted in Figure 1, to show geographic representation compared to the National TBI Cohort.

All studies were approved and overseen by their respective institution's Institutional Review Board prior to any study procedures.

### Measures

#### National TBI Cohort

A demographic questionnaire captured age, gender, race, ethnicity, and highest level of education completed. This study included only individuals indicating a history of TBI or concussion on a checklist of various health conditions. These participants also completed a questionnaire following the structure and content of the OSU-TBI, to confirm history of TBI and to classify injury severity. The OSU-TBI Worst Injury Score ranges from 1 to 5, with 1 indicating no history of TBI, 2 and 3 indicating mild TBI, 4 indicating moderate TBI, and 5 indicating severe TBI (33). These scores classified participants' injuries as mild or moderate–severe.

#### Medical Center Cohort

A demographic questionnaire captured age, gender, race (including Hispanic as an option), and highest level of education completed. History of TBI was confirmed via inclusion in a previous study requiring a diagnosis of concussion (e.g., mild TBI; Con-Tex study) or moderate–severe TBI (e.g., TBI Model Systems National Database study) or other clinical documentation as needed. Mild TBI was defined as a medical chart diagnosis of concussion with no known skull fracture or intracranial bleed; moderate–severe TBI was defined as documentation of at least one of the following: Glasgow Coma Scale score <13, loss of consciousness >30 min, post-traumatic amnesia >24 h, or positive neuroimaging.

#### Behavioral Assessment Screening Tool (BAST) in Both Cohorts

The BAST measures self-reported neurobehavioral symptoms in five domains: Negative Affect, Fatigue, Substance Abuse, Executive Function, and Impulsivity. It demonstrates good content validity and a multidimensional factor structure with good internal consistency reliabilities among community-dwelling adults with chronic TBI (30, 35). Subscale scores represent an average score for frequency of experiencing symptoms within each subscale, ranging from 1 (never) to 5 (very often).

### Analyses

We calculated frequency and percentages of demographic characteristics and means and standard deviations for each of the BAST subscales within each cohort. To address the primary aim of the present study, we descriptively compared BAST subscale scores by cohort to examine cohort differences and performed non-parametric tests for independent samples (Kruskal–Wallis, Mann–Whitney) to statistically test cohort differences. All analyses were performed using Statistical Packages for the Social Sciences (SPSS, v.24) software with a conservative overall significance level of α = 0.01 to account for multiple testing.

## Results

### Participants

#### National TBI Cohort

Of a total of *n* = 263 participants in the National TBI Cohort, *n* = 211 reported mild TBI and *n* = 52 reported moderate–severe TBI. Table 1 presents demographic and neurobehavioral symptom data for participants in the National TBI Cohort.

**Table 1:** Characteristics and neurobehavioral symptoms in two English-speaking cohorts of community-dwelling adults with TBI.

**Participant characteristics**		**National TBI cohort (*****n*** **=** **263)**		**Medical center TBI cohort (*****n*** **=** **218)**	
		**Mild TBI** ***n* = 211**	**Moderate-severe TBI** ***n* = 52**	**Mild TBI** ***n* = 85**	**Moderate-severe TBI** ***n* = 133**
		***n* (%)**	***n* (%)**	***n* (%)**	***n* (%)**
Gender	Women	114 (54.0)	20 (38.5)	52 (61.2)	51 (38.3)
	Men	92 (43.6)	30 (57.7)	33 (38.8)	82 (61.7)
	Transgender/Other	5 (2.4)	2 (3.8)	0 (0)	0 (0)
Race	White	180 (85.3)	39 (75.0)	66 (77.5)	124 (93.2)
	Black/African American	11 (5.2)	4 (7.7)	14 (16.5)	7 (5.3)
	Asian	6 (2.8)	5 (9.6)	5 (5.9)	1 (0.8)
	American Indian/Alaskan native	4 (1.9)	1 (1.9)	0 (0)	0 (0)
	Native Hawaiian/Pacific Islander	1 (0.5)	2 (3.8)	0 (0)	0 (0)
	Other	7 (3.3)	1 (1.9)	0 (0)	1 (0.8)
	Unknown	2 (0.9)	0 (0)	0 (0)	0 (0)
Ethnicity	Hispanic	23 (10.9)	6 (11.5)	4 (4.7)	3 (12.5)
	Non-Hispanic	183 (86.7)	46 (88.5)	81 (95.3)	21 (15.8)
	Unknown	5 (2.4)	0 (0)	0 (0)	109 (82.0)
Education	≤ High school	37 (17.5)	9 (17.3)	5 (5.9)	36 (27.1)
	>High school	174 (82.5)	43 (88.5)	80 (94.1)	97 (72.9)
		**Mean (*****SD*****)**	**Mean (*****SD*****)**	**Mean (*****SD*****)**	**Mean (*****SD*****)**
Age (years)		40.55 (15.50)	44.15 (15.72)	43.25 (15.68)	47.42 (14.42)
Range		18-81	21-82	21-81	21-86
BAST Subscales	Negative affect	3.26 (0.75)	3.16 (0.69)	2.69 (0.72)	2.67 (0.73)
	Fatigue	3.16 (0.89)	3.07 (0.70)	2.99 (0.83)	2.66 (0.85)
	Executive function	2.27 (0.66)	2.40 (0.68)	2.11 (0.55)	2.15 (0.59)
	Impulsivity	2.30 (0.75)	2.44 (0.82)	1.84 (0.65)	2.08 (0.74)
	Substance abuse	1.83 (0.94)	2.10 (1.03)	1.13 (0.33)	1.33 (0.66)

*All BAST Subscale values are mean (standard deviation) of the average score across items in each subscale. Data from the English-Speaking National Cohort were also published in Juengst et al. (32)*.

#### Medical Center TBI Cohort

Of the 218 participants making up the Medical Center TBI Cohort, *n* = 109 were from Study 1, *n* = 24 were from Study 2, and *n* = 85 were from Study 3. Table 1 presents demographic and neurobehavioral symptom data for participants in the Medical Center TBI Cohort.

### Neurobehavioral Symptoms Group Comparisons

The National TBI Cohort reported significantly higher scores across all BAST subscales (*p* < 0.001) than the Medical Center TBI Cohort (see Table 1). Table 2 presents neurobehavioral symptom data for participants in both cohorts broken down by gender and by educational attainment. In both cohorts, women reported higher Fatigue scores than men (*p* < 0.01). However, in the National TBI Cohort, men also reported significantly higher Substance abuse than women did (*p* = 0.001), and women reported significantly higher negative affect than men did (*p* < 0.001), which was not observed in the Medical Center TBI Cohort. In the Medical Center TBI Cohort, men reported more Impulsivity than women did (*p* = 0.010), which was not observed in the National TBI Cohort. There were no significant cohort differences in neurobehavioral symptoms by educational attainment (≥high school education vs. >high school education).

**Table 2:** Neurobehavioral symptoms by gender and education in both TBI cohorts.

	**National TBI cohort (*****n*** **=** **263)**				**Medical center TBI cohort (*****n*** **=** **218)**			
**BAST subscale**	**Mild TBI** **** ***n*** **=** **206**		**Moderate-severe TBI** **** ***n*** **=** **50**		**Mild TBI** **** ***n*** **=** **85**		**Moderate-severe TBI** **** ***n*** **=** **133**	
	**Women** ***n* = 114**	**Men** ***n* = 92**	**Women** ***n* = 20**	**Men** ***n* = 30**	**Women** ***n* = 52**	**Men** ***n* = 33**	**Women** ***n* = 51**	**Men** ***n* = 82**
Negative affect	3.42 (0.74)	3.06 (0.71)	*3*.39 (0.67)	*3*.06 (0.66)	2.68 (0.73)	2.71 (0.70)	2.83 (0.77)	2.56 (0.69)
Fatigue	3.36 (0.90)	2.91 (0.08)	3.24 (0.76)	2.98 (0.12)	3.11 (0.79)	2.81 (0.86)	2.84 (0.84)	2.55 (0.85)
Executive function	2.22 (0.67)	2.34 (0.63)	2.45 (0.71)	2.40 (0.66)	2.09 (0.51)	2.15 (0.61)	2.14 (0.57)	2.16 (0.61)
Impulsivity	2.23 (0.76)	2.37 (0.08)	2.41 (0.58)	2.53 (0.17)	1.75 (0.62)	1.98 (0.68)	1.98 (0.62)	2.14 (0.73)
Substance Abuse	1.61 (0.82)	2.08 (0.99)	1.97 (1.00)	2.20 (1.10)	1.06 (0.19)	1.23 (0.46)	1.34 (0.69)	1.33 (0.65)
**BAST subscale**	**Mild TBI** **** ***n*** **=** **211**		**Moderate-severe TBI** **** ***n*** **=** **52**		**Mild TBI** **** ***n*** **=** **41**		**Moderate-severe** ** TBI** ***n*** **=** **177**	
	**≤HS** **** ***n*** **=** **37**	**>HS** **** ***n*** **=** **174**	**≤HS** **** ***n*** **=** **9**	**>HS** **** ***n*** **=** **43**	**≤HS** **** ***n*** **=** **5**	**>HS** **** ***n*** **=** **36**	**≤HS** **** ***n*** **=** **80**	**>HS** **** ***n*** **=** **97**
Negative affect	3.32 (0.71)	3.25 (0.75)	3.32 (0.80)	3.12 (0.68)	2.86 (1.14)	2.68 (0.69)	2.82 (0.80)	2.61 (0.70)
Fatigue	3.14 (0.89)	3.16 (0.90)	3.22 (0.62)	3.04 (0.72)	3.30 (1.31)	2.97 (0.79)	2.74 (0.91)	2.63 (0.79)
Executive function	2.39 (0.64)	2.24 (0.66)	2.52 (0.64)	2.37 (0.69)	2.18 (0.74)	2.11 (0.54)	2.22 (0.64)	2.12 (0.57)
Impulsivity	2.31 (0.79)	2.29 (0.75)	2.31 (0.54)	2.47 (0.87)	2.05 (0.41)	1.83 (0.66)	2.13 (0.69)	2.06 (0.76)
Substance abuse	1.83 (1.01)	1.83 (0.92)	2.15 (1.03)	2.09 (1.05)	1.07 (0.15)	1.13 (0.34)	1.49 (0.95)	1.27 (0.52)

*All values are mean (standard deviation) of the average score across items in each subscale. Individuals identifying as Transgender/Other for gender are not included in the table due to very small sample size when breaking down by injury severity (n = 5 mild TBI; n = 2 moderate-severe TBI) in the National TBI Cohort and no individuals identifying as Transgender/Other in the Medical Center TBI Cohort*.

## Discussion

We identified symptom differences between two cohorts of individuals with TBI recruited from different sampling frames, demonstrating that individuals with TBI recruited through academic and clinical institutions may not be representative of individuals with TBI living across the United States. Across all symptom domains, those in the nationally representative sample reported more frequent neurobehavioral symptoms than those in the sample recruited through academic and clinical institutions. Racial and ethnic differences and geographic location as a proxy indicator of access to quality healthcare systems may explain the cohort differences we observed (14). Individuals with TBI living further from major medical centers not only have more pre- and post-injury comorbities, which can contribute to neurobehavioral symptoms (32), but also report more unmet service needs (28), suggesting that their underlying conditions are not adequately managed. In addition to the broader geographic representation in our National TBI Cohort, there was also a greater racial and ethnic diversity compared to our Medical Center Cohort. Prior work on racial and ethnic disparities after TBI suggests that factors associated with access to healthcare, including socioeconomic status, quality of education, access to care, quality of care, and transportation barriers (14), may explain differences in post-TBI functional outcomes.

We anticipated that educational attainment would partially explain these cohort differences, based on past literature (36–38), but we found no differences in neurobehavioral symptoms by educational attainment in either cohort, and on average, participants in both cohorts had relatively comparable rates of post-secondary education. However, there were differences in educational attainment when breaking each cohort down by injury severity. Those in the National Cohort who had a mild TBI were far less likely to have any post-secondary education than those in the Medical Center Cohort with a mild TBI, but those with a moderate–severe TBI in the National Cohort were more likely to have post-secondary education than those with moderate–severe TBI in the Medical Center Cohort. The potential protective effects of education may partially explain why those in the Medical Center Cohort with mild TBI reported fewer symptoms. More vulnerable individuals (e.g., lower education, lacking insurance) from minority groups may not recognize the need for medical care after a mild TBI or may not be able to allocate the limited resources available to them to address symptoms they view as mild (21). For those with a history of more severe injuries, the cognitive consequences of TBI may overshadow the protective effects of post-secondary education on neurobehavioral symptoms. For them, high-quality healthcare—especially early after moderate–severe TBI—may be a more important factor. Racial and ethnic minorities are less likely to receive inpatient rehabilitation after TBI (17), and when they do, the time to rehabilitation admission is longer compared to non-Hispanic white patients (21). Therefore, the more frequent neurobehavioral symptoms in the National Cohort participants with moderate–severe TBI, despite a greater proportion having completed post-secondary education, may be a result of differential healthcare access, quality, and utilization.

Similar to past literature on gender differences in symptom reporting after TBI (12, 13), we found that women reported more fatigue symptoms than did men, and men reported more impulsivity and substance abuse symptoms than did women. However, other than the finding that women reported more fatigue than men did, these gender differences were not consistent across the two cohorts. The extent to which this is attributable to men representing a larger proportion of those with moderate–severe TBI vs. mild TBI and/or to the National TBI cohort including a substantially larger proportion of mild TBI than moderate–severe TBI remains unclear. Therefore, though notable cohort differences exist, the effect of the interaction between gender and cohort on neurobehavioral symptoms after TBI requires further study.

Comparing these two cohorts revealed the limitations inherent in the recruitment methods and catchment areas for a substantial proportion of the research published on chronic TBI, particularly with regard to representativeness. The Medical Center Cohort had a higher percentage of participants who were white, non-Hispanic, and, for those with mild TBI, had some post-secondary education. Though it represented participants recruited from two academic medical centers and two clinical rehabilitation centers across three geographically separated metropolitan areas in the United States, it did not have the same geographic coverage as the National TBI Cohort. Since the BAST yielded higher scores overall in the National TBI Cohort than the Medical Center Cohort, we strongly urge that meaningful within-person change scores, rather than hard cutoff scores, be established for clinical interpretation. Within-person change would be less prone than hard cutoff scores to external factors (e.g., access to healthcare, quality of education, etc.) that are difficult to quantify and adjust for in norm-based scores. Establishing within-person symptom variability at the individual level and defining meaningful change as that which falls outside of natural variability would most effectively account for the multitude of unmeasurable factors contributing to neurobehavioral symptoms. Future research should identify novel approaches to ensure valid and meaningful measurement of long-term outcomes and should identify modifiable barriers to quality care after TBI for individuals who are most vulnerable to healthcare disparities.

### Limitations

The Medical Center Cohort comprised three distinctly different studies, with different inclusion and exclusion criteria, aims, and data collection methods, potentially introducing additional bias related to sample selection. Furthermore, we did not collect data on important factors that may explain differences in the two cohorts, including participants' access to healthcare, health and treatment history, and social support systems. Though we took multiple steps to ensure the validity of all data, data collected via anonymous survey are prone to bias and error. Most notable were differences between the two cohorts in identification, confirmation, and severity classification of TBI. While the Medical Center Cohort relied on medical documentation, the National Cohort relied on self-report via an electronic structured version of the OSU TBI asking questions about history of head injury via a variety of mechanisms, loss of consciousness (presence and duration), and experiencing a period of feeling dazed or confused after injury. Confirmation of injury and characterization of severity are determined by study investigators based on answers to these questions, following structured guidelines and scores for the OSU TBI (33, 34). However, despite efforts to ensure validity of self-reported TBI, differences in methodology between the cohorts introduces potential bias. Finally, the Medical Center Cohort included a larger proportion of individuals with moderate to severe TBI than the National Cohort, which was predominantly mild TBI. In the absence of measures to characterize cognitive ability and self-awareness, we cannot say with certainty whether the differences in self-reported symptoms are due to different symptom patterns based on injury severity, self-awareness differences (e.g., those with milder injuries may have better self-awareness of the symptoms they are experiencing), or other factors outside of injury that differed between cohorts.

## Conclusions

Individuals with TBI connected to academic or clinical rehabilitation centers may systematically differ from the broader population of adults with TBI across the United States. Disparities in access to and utilization of healthcare services may contribute to more frequent and untreated neurobehavioral symptoms. Clinicians and researchers should take care when generalizing results from studies with non-representative samples, especially when establishing cutoff scores for patient-reported outcomes.

## Data Availability Statement

The datasets generated for this study are available on request to the corresponding author.

## Ethics Statement

The studies involving human participants were reviewed and approved by University of Texas Southwestern Medical Center, Baylor Scott & White Institute for Rehabilitation, and University of Pittsburgh. Participants provided informed consent to participate in these studies.

## Author Contributions

SJ was the senior author, principal investigator on two of the studies presented in this manuscript, and co-investigator on the two other studies presented. She was the primary author responsible for writing, conceptualization, and final decisions. AN contributed to data preparation, conducted extensive literature review, and drafted significant portions of the manuscript. LT collaborated with SJ in the initial study conceptualization, provided consultation on all statistical analysis as a statistician, and drafted significant portions of the methods and results. All authors have made significant contributions to the conceptualization, interpretation, writing of this manuscript, and the study described herein and have read and approved the final manuscript.

### Conflict of Interest

The authors declare that the research was conducted in the absence of any commercial or financial relationships that could be construed as a potential conflict of interest.
